# Point‐of‐care breath sample analysis by semiconductor‐based E‐Nose technology discriminates non‐infected subjects from SARS‐CoV‐2 pneumonia patients: a multi‐analyst experiment

**DOI:** 10.1002/mco2.726

**Published:** 2024-10-24

**Authors:** Tobias Woehrle, Florian Pfeiffer, Maximilian M. Mandl, Wolfgang Sobtzick, Jörg Heitzer, Alisa Krstova, Luzie Kamm, Matthias Feuerecker, Dominique Moser, Matthias Klein, Benedikt Aulinger, Michael Dolch, Anne‐Laure Boulesteix, Daniel Lanz, Alexander Choukér

**Affiliations:** ^1^ Department of Anesthesiology LMU University Hospital Ludwig Maximilian University Munich Germany; ^2^ Institute for Medical Information Processing Biometry and Epidemiology Faculty of Medicine Ludwig Maximilian University Munich Germany; ^3^ Munich Center for Machine Learning Munich Germany; ^4^ LANZ GmbH Bergisch Gladbach Germany; ^5^ Airbus Defence and Space GmbH Claude‐Dornier‐Straße Immenstaad Germany; ^6^ Emergency Department LMU University Hospital Ludwig Maximilian University Munich Germany; ^7^ Department of Medicine II LMU University Hospital Ludwig Maximilian University Munich Germany; ^8^ Department of Anesthesiology Inn Klinikum Altötting Germany

**Keywords:** breath gas, COVID‐19, E‐Nose, machine learning, mass spectrometry, metal oxide sensor, pneumonia, volatile organic compounds

## Abstract

Metal oxide sensor‐based electronic nose (E‐Nose) technology provides an easy to use method for breath analysis by detection of volatile organic compound (VOC)‐induced changes of electrical conductivity. Resulting signal patterns are then analyzed by machine learning (ML) algorithms. This study aimed to establish breath analysis by E‐Nose technology as a diagnostic tool for severe acute respiratory syndrome coronavirus type 2 (SARS‐CoV‐2) pneumonia within a multi‐analyst experiment. Breath samples of 126 subjects with (*n* = 63) or without SARS‐CoV‐2 pneumonia (*n* = 63) were collected using the ReCIVA® Breath Sampler, enriched and stored on Tenax sorption tubes, and analyzed using an E‐Nose unit with 10 sensors. ML approaches were applied by three independent data analyst teams and included a wide range of classifiers, hyperparameters, training modes, and subsets of training data. Within the multi‐analyst experiment, all teams successfully classified individuals as infected or uninfected with an averaged area under the curve (AUC) larger than 90% and misclassification error lower than 19%, and identified the same sensor as most relevant to classification success. This new method using VOC enrichment and E‐Nose analysis combined with ML can yield results similar to polymerase chain reaction (PCR) detection and superior to point‐of‐care (POC) antigen testing. Reducing the sensor set to the most relevant sensor may prove interesting for developing targeted POC testing.

## Graphical abstract

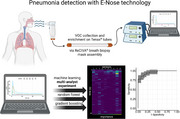

## 1 INTRODUCTION

Volatile organic and non‐organic compounds (VOC) such as alcohols, aldehydes, and ketones in exhaled breath provide an insight into the metabolic processes taking place in the human body. VOC are emitted by all cells, and differ in their specific composition depending on the type of cell and its current metabolism.^1^ Thus, different physiological states can affect VOC composition, reflecting food intake, lifestyle, or exercise.^2^, ^3^ VOC analysis is a growing field with many possible applications, including diagnostic approaches in medicine.^4^ For example, VOC analysis allowed for differentiation between bacteria species in vitro^5^ and in vivo,^6^ and for detection of influenza virus in swine.^7^ Even different influenza virus subtypes could be discriminated in an infected cell line based on the emitted VOC signature.^8^ In addition, VOC have been discussed to potentially identify patients with metabolic diseases such as diabetes,^9^ with malignant diseases such as neck, bladder, colon, or lung cancer,^9^, ^10^, ^11^, ^12^ and with lung diseases such as chronic obstructive pulmonary disease or asthma.^13^, ^14^ With regard to the severe acute respiratory syndrome coronavirus type 2 (SARS‐CoV‐2) pandemic, primary studies show that SARS‐CoV‐2 pneumonia patients could be distinguished from non‐infected subjects using VOC‐based breath analysis.^15^, ^16^, ^17^

Thus far, mass spectrometry is the method of choice for VOC analysis.^16^ However, high acquisition and running costs as well as the need for laboratory support show its limitations. In contrast, metal oxide sensor‐based electronic nose (E‐Nose) technology provides a cheaper, portable, and easy to use method for breath analysis, especially in point‐of‐care (POC) environments. Unlike mass spectrometry, E‐Nose technology does not identify single compounds, but detects VOC‐induced changes of electrical conductivity in a sensor set of metal oxide semiconductors. These changes of conductivity generate a signal pattern for each sample, which is then analyzed by machine learning (ML) algorithms. Various setups of electronic noses with different types of sensor sets have been applied for breath research, for example, sensor arrays of conducting polymers, nanomaterial‐based or quartz microbalance‐based sensors, colorimetric sensors, and metal oxide sensors. Electrical properties of the metal oxide semi‐conductors can be optimized for different VOC detection by adjusting sensitivity through altering sensor film thickness or loading of the metal oxide surface with noble metal.^18^ E‐Nose technology applied in this study represents a technology transfer from space to health. Previous E‐Nose experiments have been conducted to successfully determine bacterial surface contamination on the International Space Station,^19^ and the technique has now been adapted and optimized for its use in a medical setting.

With SARS‐CoV‐2 being the predominant cause for pneumonia during the pandemic, this study aimed to establish breath analysis by E‐Nose technology as a diagnostic tool in SARS‐CoV‐2 infection. The particularity of the present study is that it was conducted as a so‐called multi‐analyst experiment.^20^, ^21^, ^22^, ^23^ More specifically, after an optimized pre‐analytical sampling method was established, the resulting data was further analysed by three independent teams of data analysts who applied different ML approaches, without consulting each other. This still uncommon multi‐analyst approach was inspired by Wagenmakers et al., who state that “one statistical analysis must not rule them all”,. and “a single analysis hides an iceberg of uncertainty” while “multi‐team analysis can reveal it”.^23^ It is established that the high multiplicity of possible analysis strategies resulting from these uncertainties, if combined with selective reporting, can substantially contribute to the so‐called replication crisis in science,^24^ including in the context of ML. The rapidly growing field of artificial intelligence is unfortunately not immune against irreproducibility issues.^25^ Multi‐analyst approaches are known to strengthen the robustness of results and conclusions obtained from analysis of datasets^26^ and to show that analytical flexibility can have substantial effects on scientific conclusions.^20^ Thus, results obtained by the three teams, featuring a number of common aspects but also differences, allow us to formulate more reliable results than a single analysis would do. At the same time, it illustrates the huge uncertainty related to analytical choices and its impact on the results—a still largely unexplored issue in the field of artificial intelligence application in medicine.

## 2 RESULTS

### 2.1 Patients

From May 2020 until November 2021, 63 patients with PCR‐confirmed SARS‐CoV‐2 infection and 63 subjects without infection were enrolled in this study. Patients and uninfected subjects did not differ with regard to their age, sex, body mass index, or smoking habit (Table 1). Diagnosed comorbidities showed no statistical difference between patients and controls (Table S1).

**TABLE 1:** Characterization of hospitalized patients with severe acute respiratory syndrome coronavirus type 2 (SARS‐CoV‐2) infection and of non‐infected controls.

	COV (*n* = 63)	CTRL (*n* = 63)	*p*‐Value
Age (years)	53 (48‒65)	56 (46‒63)	0.705^a^
Female sex (%)	19 (30.16)	19 (30.16)	1.0^b^
BMI (kg/m^2^)	28.53 (25.21‒31.41)	26.81 (24.38‒30.44)	0.369^a^
Smoker (%)	3 (4.76)	8 (12.70)	0.117^b^
Days			
In hospital	7 (5‒12)		
In ICU	9.5 (6.25‒14.5)		
Patients in ICU (%)	12 (19)		
Day of admission to ICU	3.5 (3‒5)		
Respiratory rate	20 (18‒23.5)	14 (10‒16)	<0.001^a^
SpO_2_	95 (93‒96)		
Hemoglobin (g/dL)	13.8 (12.6‒14.6)	*11.5*‒*17.5*	
Erythrocytes (T/L)	4.69 (4.31‒5.04)	*3.96*‒*5.77*	
Thrombocytes (G/L)	197 (142‒258)	*146*‒*391*	
Leukocytes (G/L)	5.32 (4.34‒6.31)	*3.90*‒*10.40*	
Lymphocytes (G/L)	1.075 (0.76‒1.39)	*1.05*‒*3.56*	
(%)	20 (14‒25.25)	*18*‒*48*	
Monocytes (G/L)	0.42 (0.26‒0.63)	*0.25*‒*0.87*	
(%)	7 (5‒10)	*4*‒*15*	
Neutrophils (G/L)	3.69 (2.925‒4.71)	*1.78*‒*7.37*	
(%)	70 (61.5‒78.5)	*40*‒*71*	
C‐reactive protein	5.55 (2.5‒8.15)	*<0.5*	
IL‐6	15.47 (9.41‒26.99)	*<5.9*	

*Note*: Italic font indicates reference range. Values represent median (interquartile range).

Abbreviations: BMI, body mass index; COV, coronavirus disease‐2019 patients; CTRL, controls; ICU, intensive care unit; IL, interleukin.

^a^ For statistical analyses, Mann–Whitney *U*‐test was applied.

^b^ For statistical analyses, Fisher's exact test was applied.

Upon admission, patients presented with a median peripheral oxygen saturation (SpO_2_) of 95% on room air. Blood analyses revealed slightly elevated levels of C‐reactive protein, while median leukocyte counts were within reference range. Median hospitalization time on the normal ward was 7 days. Out of 63 patients, 12 patients subsequently required intensive care unit (ICU) treatment, with a median ICU stay of 9.5 days (Table 1). Two fatal outcomes were observed during the evaluated period.

Breath samples were measured as described in Section 4. Raw data of signal patterns was subjected to analyses by three independent parties, designated teams A, B, and C. The total set of seven experiments is numbered consecutively from I to VII.

### 2.2 Data analyses by three independent parties

The three teams differ in their general approaches from data pre‐processing to the interpretation of the results. Team B and C's main goal was to fully optimize the classification error and other performance measures using a variety of sophisticated methods such as gradient boosting (team B) or multi‐layer perceptron (team C). Team A aimed to enhance general predictability and conservation of interpretability by applying more intrinsic interpretation methods instead of model‐agnostic approaches. The chosen learners (team A: random forest [RF], team B: gradient boosting, team C: extra tree [ET], decision tree [DT], RF) differed considerably in the construction of their underlying prediction function ‐ a phenomenon called Rashomon effect.^27^, ^28^

In the following, results of the experiments conducted by the three teams are described in detail.

### 2.3 Team A

#### 2.3.1 Experiment I: time‐independent classifier exploration and variable importance

**FIGURE 1:**
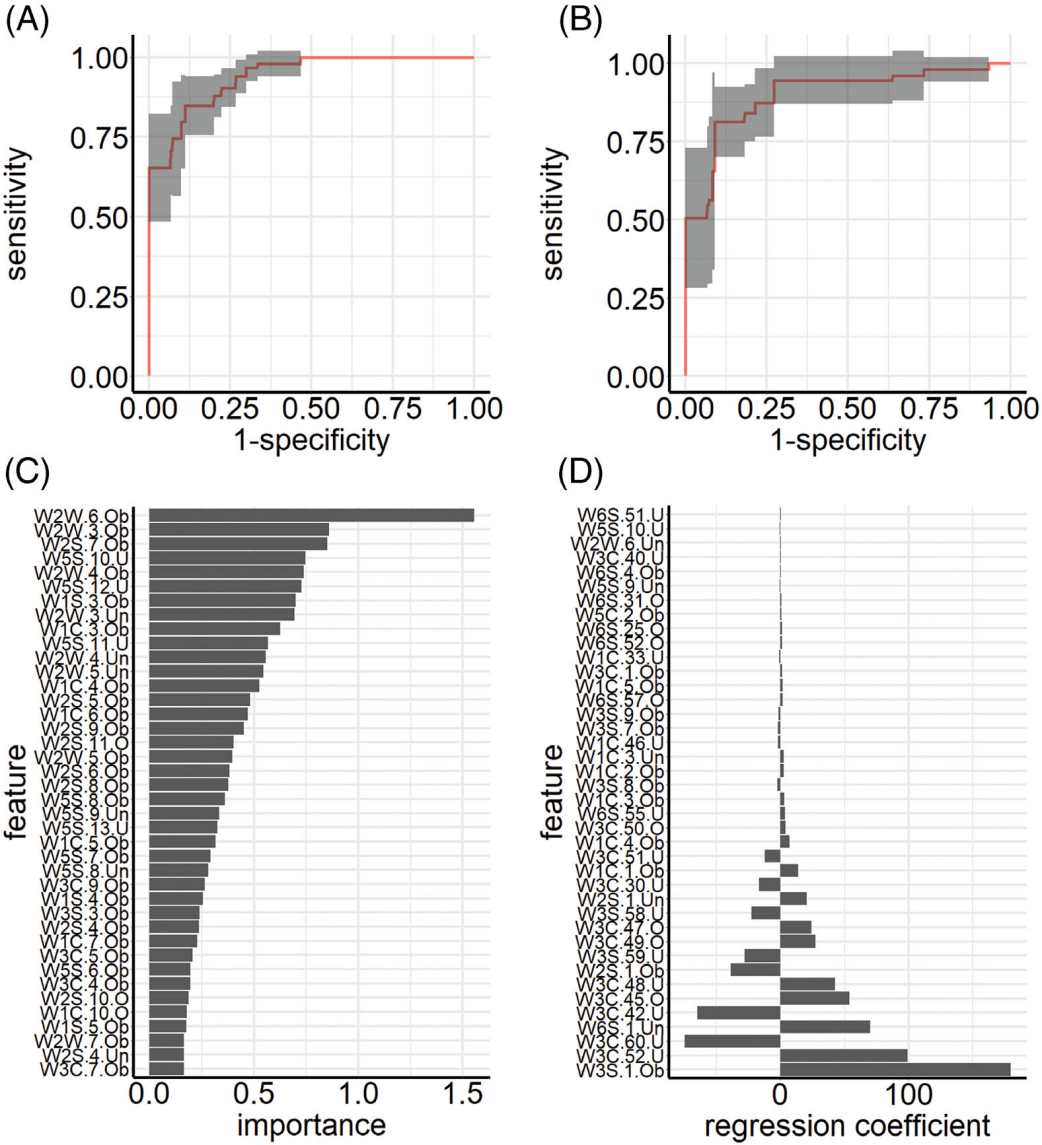
Team A, experiment I: ROC curves and feature importance. ROC curves depicting the sensitivity (*y*‐axis) and 1 − specificity (*x*‐axis) for the random forest (RF) (A, AUC: 0.94) and Glmnet classifier (B, AUC: 0.90). The bold line shows the micro‐averaged ROC curve based on the resampling results with its pointwise confidence interval. Panel (C) depicts variable importance (mean decrease in impurity) for the top 40 features of the RF classifier and panel (D) shows regression coefficients for the top 40 features of the Glmnet classifier.

Experiment I by team A does not explicitly make use of the time structured data, resulting in a typical p > > n (“high—p, low—n”) problem, meaning that the model receives more predictors per subjects than subjects themselves. Figure 1A,B depicts the averaged receiver operating characteristic (ROC) curves for the RF and Glmnet classifier, respectively. Note that the displayed naive confidence intervals might underestimate the true underlying uncertainty due to correlation of the cross‐validation iterations, and thus only reflect the order of magnitude of the variability. The RF classifier has an average misclassification error (ME) of 0.13 (AUC: 0.94). The Glmnet classifier performs slightly worse with an average ME of 0.15 (AUC: 0.90). Figure 1C shows the variable importance based on the mean decrease in impurity for the RF classifier. The features with the highest predictive power are mainly features from the upper respiratory tract within the first 10 s of the measurement. This makes sense as the highest variability of the raw measurements can be seen within this period.

Figure 1D depicts the regression coefficients of the Glmnet classifier. The results are not as clear‐cut as for classifier RF. Features with the highest impact on the prediction results do not only stem from different sensors but also different time points of measurements. The Glmnet classifier focuses more on later time points of each sensor.

#### 2.3.2 Experiment II: time‐dependent feature extraction and variable importance

**FIGURE 2:**
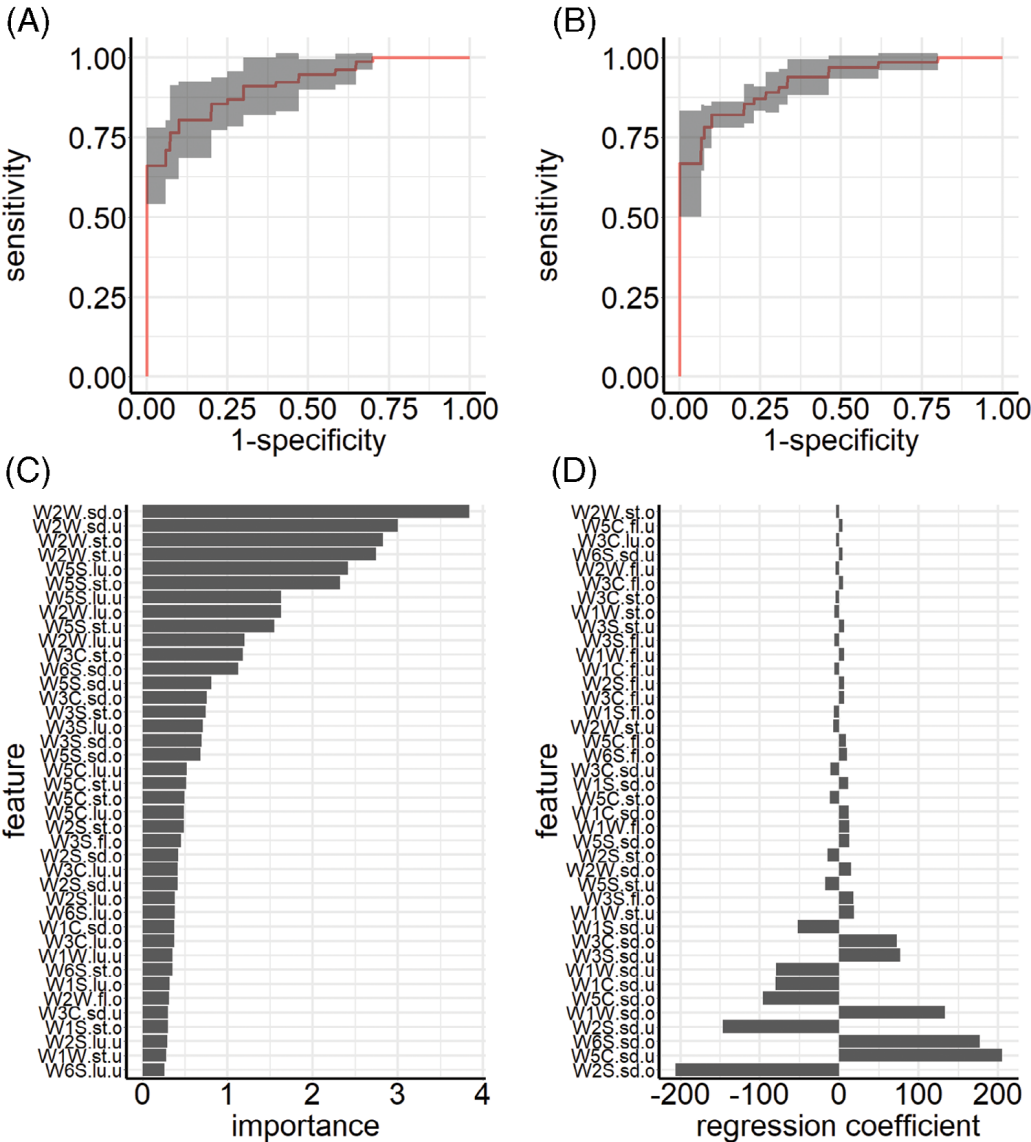
Team A, experiment II: ROC curves and feature importance. Performance of random forest (RF) (A, AUC: 0.91) and Glmnet (B, AUC: 0.92) classifier on data generated via feature extraction. The micro‐averaged ROC curve is depicted (bold line) with the corresponding confidence interval (gray). The impact of different generated features on classification accuracy is shown through variable importance for the RF classifier (C), and regression coefficients for the Glmnet classifier (D).

The second experiment by team A makes use of feature extraction techniques, also termed “dimensionality reduction.” Figure 2A,B depicts the averaged ROC curves for the RF and Glmnet classifier, respectively. The RF classifier has an average misclassification rate of 0.19 (AUC: 0.91). The Glmnet classifier performs better with an average misclassification rate of 0.13 (AUC: 0.92). Figure 2C illustrates the variable importance based on the mean decrease in impurity for the RF classifier. Features with the highest predictive power are features from both upper and lower respiratory tracts. Especially sensor 9 seems to have a high influence on the predictive performance of the model. Regression coefficients of the Glmnet classifier are depicted in Figure 2D, where results again differ from the RF classifier. The Glmnet classifier focuses on both the standard deviations of the upper and lower respiratory tracts for most sensors.

### 2.4 Team B

#### 2.4.1 Experiments III and IV: feature extraction and hyperparameter tuning on all sensors

**FIGURE 3:**
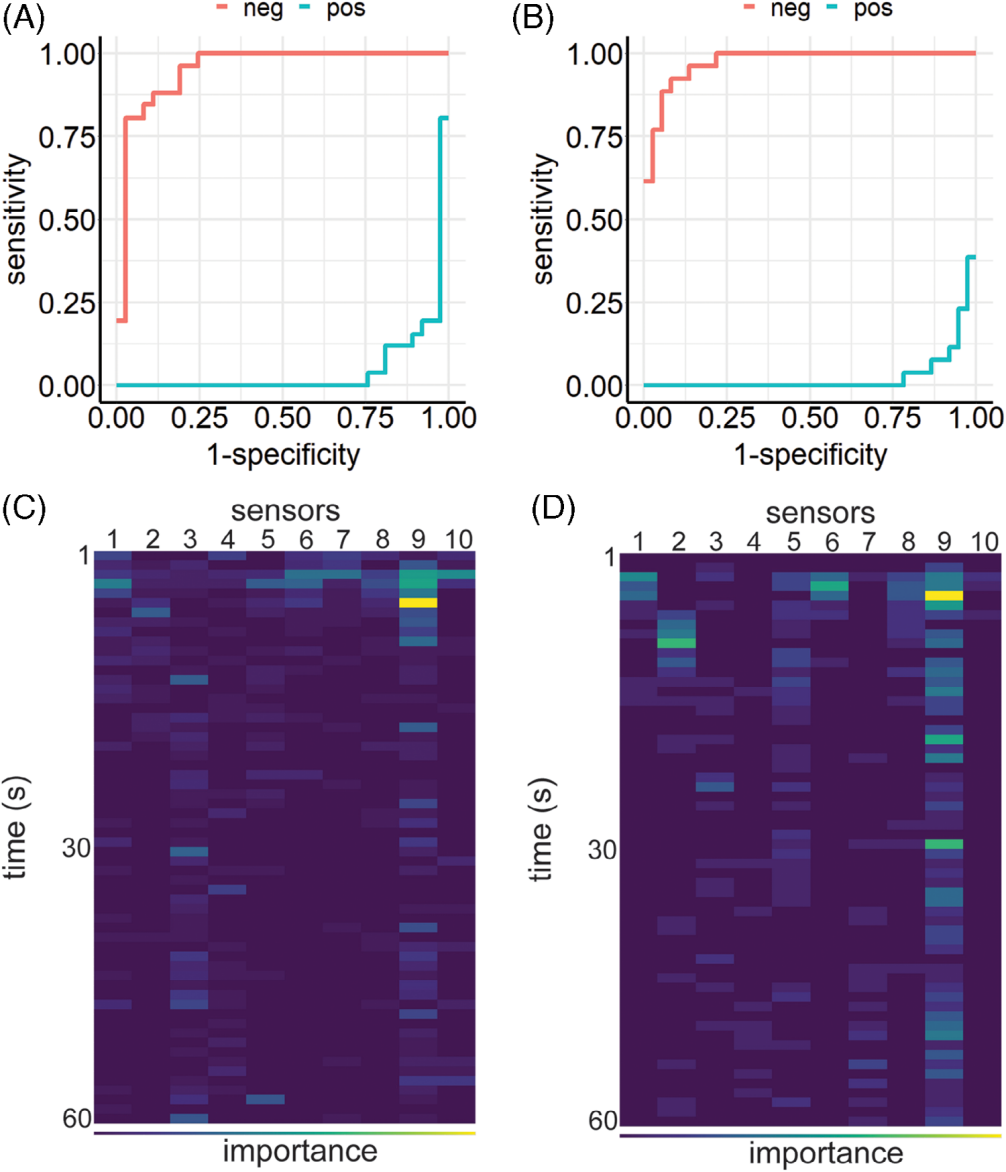
Team B: ROC curves and importance maps for experiments III and IV. ROC curves (A and B) represent the performance of the learner on data from all sensors. Comparing classification impact of features on importance maps shows that sensor 9 has the highest importance for classification (C and D). While experiment III (A and C) obtained results without hyperparameter tuning, experiment IV (B and D) depicts performance of the model with hyperparameter tuning.

These experiments used LightGBM's gradient boosting model and the approach described above. Without hyperparameter optimization, the learner achieved an overall performance of ME = 0.143 (Figure 3A). Combined with Optuna's hyperparamter optimization the learner improved to ME = 0.079 (Figure 3B). F1 results and confusion matrices are provided in Figure S1. Sensor 9 showed the highest importance for classification (Figure 3C,D).

#### 2.4.2 Experiments V and VI: feature extraction and hyperparameter tuning on sensor 9

**FIGURE 4:**
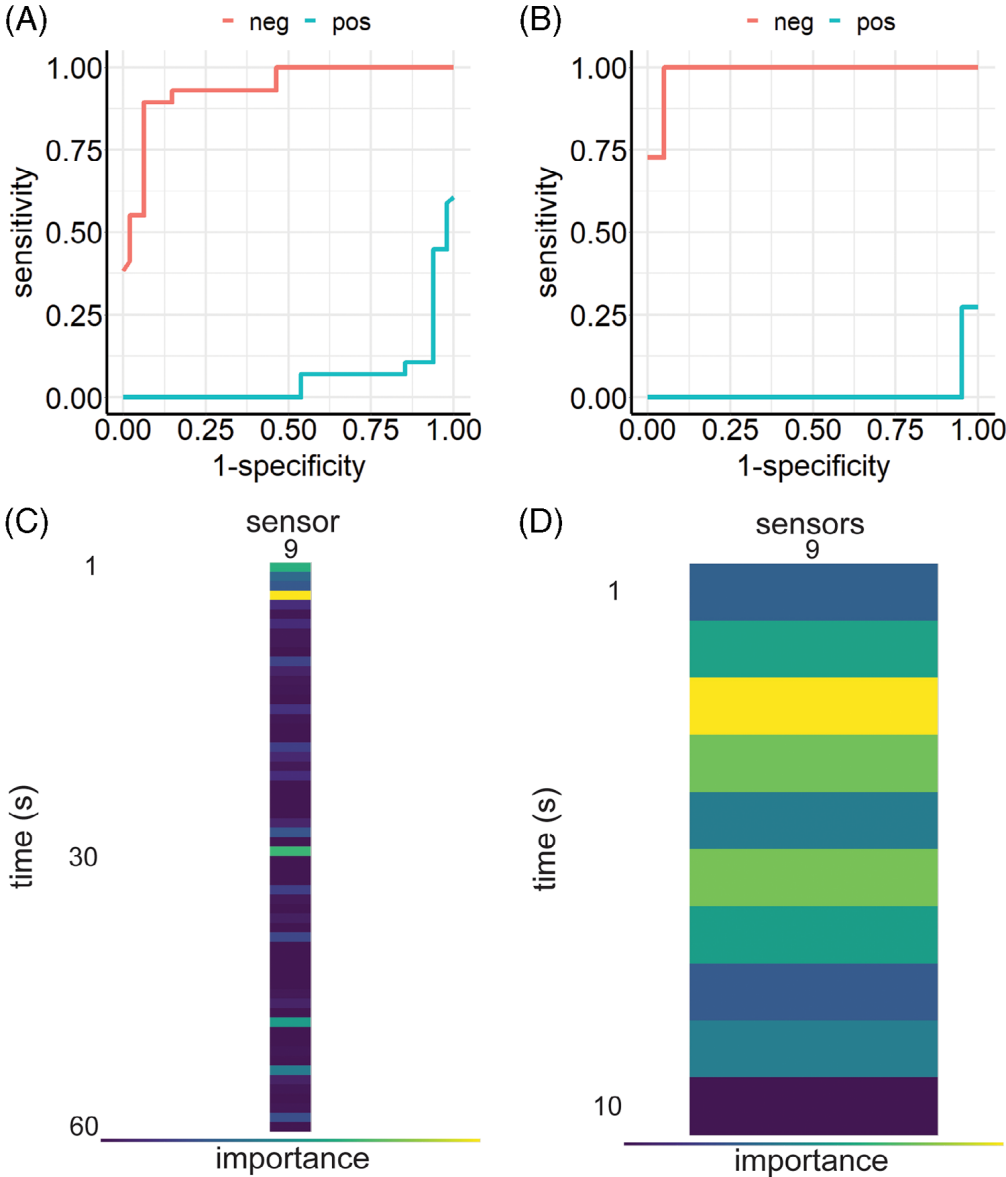
Team B: ROC curves and importance map for experiments V and VI. ROC curves show results when the learner was only provided with data from sensor 9 without (A) or with (B) hyperparameter tuning. As for experiments III and IV, the highest impact can be observed during the initial phase of measurements (C, without hyperparameter tuning; D, with hyperparameter tuning), corresponding to peak signals of raw data (see Figure 6).

The importance of sensor 9 led to an additional analysis of this specific sensor alone, where only data generated by sensor 9 was used. LightGBM's gradient boosting model achieved a ME of 0.104 (Figure 4A). Figure 4C depicts time dependent importance of sensor 9 measurements, showing that the first seconds have the highest impact on classification. Thus, subsequent Optuna's hyperparameter optimization was performed on filtered raw data, where only the first 10 s of sensor 9 measurements were used. The train test split was set to 9:1. This resulted in a performance of ME = 0.065, outperforming all previous models (Figure 4B,D). Figure S2 shows F1 results and confusion matrices, respectively.

### 2.5 Team C

During preliminary testing for approach C, similar results were obtained for three out of four classifiers (ET, DT, RF): for both upper and lower respiratory tract, sensor 9 showed the best classification quality (Figure S3). Variance of classification with regard to hyperparameters was also smallest for this sensor. Classifier RF showed the best results of all classifiers, and sensor 9 measurements from lower respiratory tract samples provided the best results (Figure S3). Thus, it was decided to continue the investigation with classifier RF.

Establishing the ground truth for classifier RF showed not all subjects were identified correctly, although the score of false positives in both cohorts was much lower than during the real leave‐one‐out test (Table S2 and Figure S3). Sensor 9 again showed superior performance regarding hyperparameter sensitivity (Figure S4). Thus, the following steps were performed with classifier RF and sensor 9 only.

#### 2.5.1 Experiment VII: influence of prevalence and training data on classifier RF and sensor 9

Using a RF classifier with hyperparameter tuning on sensor 9 measurements from lower respiratory tract samples resulted in an overall performance of ME = 0.062. A superior hyperparameter combination could be identified for classifier RF and sensor 9. Table 2 shows the best values for sensitivity and specificity achieved by the classifier.

**TABLE 2:** Experiment VII: sensitivity P(+|C) and specificity P(‒|nC) for the best hyperparameter combination with classifier random forest (RF) for upper (A) and lower (B) respiratory tract.

Sensitivity upper respiratory tract P(+|C) | (A)	Sensitivity lower respiratory tract P(+|C) | (B)	Specificity upper respiratory tract P(‒|nC) | (A)	Specificity lower respiratory tract P(‒|nC) | (B)
0.94915	0.96610	0.89552	0.91045

*Note*: Values for the best hyperparameter combination for classifier RF and sensor 9.

**FIGURE 5:**
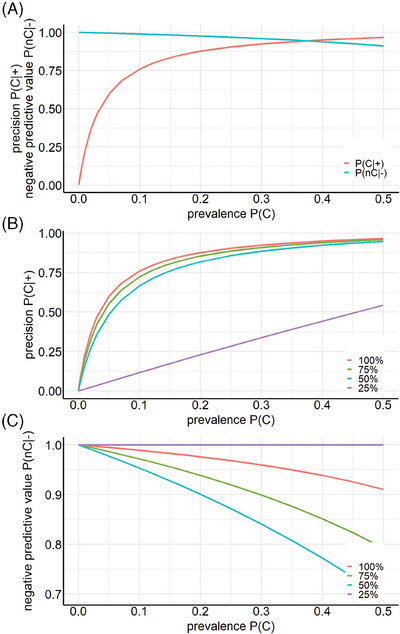
Team C, experiment VII: precision P(C|+) and negative predictive value P(nC|‒) versus prevalence P(C). (A) Performance of random forest (RF) learner on data of sensor 9 from lower respiratory tract samples is dependent on the prevalence of severe acute respiratory syndrome coronavirus type 2 (SARS‐CoV‐2) in the test population. (B and C) Performance of the model is also dependent on the amount of training data provided to the learner during training. Depicted are precision (B) and negative predictive value (C) versus prevalence from sensor 9 data obtained from lower respiratory tract samples.

Precision and negative predictive value depend on the probability of encountering SARS‐CoV‐2 in a test population (prevalence, *P*(*C*)). Sensitivity and specificity of the best classifier were determined for values of *P*(*C*) = 50%, as there were 63 subjects for each label. *P*(*C*) is necessarily variable in the real world. Figure 5A shows how precision and negative predictive value depend on *P*(*C*) in the group to be examined.

Table 3 depicts the true/false positive/negative rates as a function of the amount of training data used. At 25%, only 10 out of 63 non‐infected subjects were correctly identified as non‐infected, all others, including all SARS‐CoV‐2 subjects, were classified SARS‐CoV‐2. This poor classification leads to a constant value of “1” for the negative predictive value because all subjects that were identified as uninfected are uninfected, but only 10 out of 63 were correctly classified as uninfected. For larger amounts of training data, precision and negative predictive values increased with increasing amount of training data (Figure 5B,C).

**TABLE 3:** Experiment VII: true/false positive/negative rates versus amount of training data.

Amount of training data (%)	P(+|C)	P(+|nC)	P(‒|C)	P(‒|nC)
100	0.9047619	0.03174603	0.0952381	0.96825397
75	0.7460317	0.03174603	0.25396825	0.96825397
50	0.5714286	0.03174603	0.42857143	0.96825397
25	1	0.84126984	0	0.15873016

*Note*: True positive P(+|C), false positive P(‒|C), true negative P(‒|nC), and false negative P(+|nC) rates are depicted as a function of the amount of training data used. At 25%, only 10 out of 63 non‐infected subjects were correctly identified as non‐infected, all others, including all severe acute respiratory syndrome coronavirus type 2 (SARS‐CoV‐2) subjects, were classified as SARS‐CoV‐2.

#### 2.5.2 Experimentation with deep learning models

In the multilayer perceptron model, sensors 2 and 9 performed best although the score was worse than with the previous classifiers and the variation of hyperparameters was larger (Figure S5A,B). Therefore, this process was repeated with slightly adapted hyperparameter ranges and 50 variations for sensors 2 and 9 only (Figure S5C,D). In comparison to the more classical ML approach, the multilayer perceptron could not outperform the RF classifier on sensor 9 measurements from lower respiratory tract samples.

## 3 DISCUSSION

In this study, a rapid, portable, and cheap breath gas analysis protocol was established. Exhaled VOC were measured by an array of ten metal oxide semiconductor sensors and changes in conductivity were recorded.

Raw data were subjected to three independent ML approaches, including RF and gradient boosting tree learners. The multi‐analyst experiment shows that E‐Nose technology in combination with the presented novel sampling method allows for successful identification between SARS‐CoV‐2‐positive patients and uninfected volunteers with MEs ranging from 0.06 to 0.19. Sensitivity and specificity reached values of about 90% or higher for all three independent teams, underlining the robustness of the data generated.

The teams’ approaches already showed differences in their data pre‐processing strategies. While team A analyzed the data both without taking into account time dependencies between features or by taking them into account through a feature extraction procedure, teams B and C did not use feature extraction but different approaches to reduce the number of features manually before training. It is interesting that even if simple variance and min/max measures are extracted from the original time series (team A) or if raw data are reduced drastically to the first 10 s of the measurement and/or only one sensor (team B), the differences in performances are small. Overall, it can be concluded that despite these major differences between the three analytical approaches and their rationales, all results were in a decent range in terms of ME (0.06–0.19). This indicates that this study was successful in demonstrating predictability of the underlying system.

The most substantial difference between the three teams’ results lies in the interpretation of the contributions of the features/sensors to the prediction models’ performance, which is not as clear‐cut. All three teams identify sensor 9 and the first seconds of the time series measurement as most informative. However, other model interpretations are conflicting and conclusions cannot be easily drawn without more domain knowledge on the respective sensors. This can be observed in particular if we compare the results of the model‐agnostic interpretation methods to the natively interpretable regression coefficients obtained by team A, that show a completely different picture of the importance of the different features.

Highly accurate testing methods for SARS‐CoV‐2 have been the focus of several studies. Currently available and widely used antigen tests (Ag‐RDT) can reach a sensitivity of up to 81.9% and a specificity of up to 98.9%.^29^, ^30^ More expensive and less widely available, PCR testing has been shown to be superior to antigen testing with a sensitivity and specificity of up to 92.8% and 97.6%, respectively.^30^ However, accuracy of PCR results vary. Up to 54% of SARS‐CoV‐2‐infected patients may initially obtain a false‐negative RT‐PCR result,^31^, ^32^, ^33^, ^34^ and over 60% of errors occur in the preanalytical phase of any diagnostic process.^32^ These reports indicate that breath gas analysis by E‐Nose technology as demonstrated here may be superior to commercially available antigen tests and comparable to PCR testing.

Many studies focused on the identification of specific VOC possibly serving as biomarkers for SARS‐CoV‐2 infection.^15^, ^35^ In contrast, the approach chosen here aimed at allocating patterns of VOC‐induced changes in conductivity of a sensor array, generating a breath gas fingerprint of each subject. Since several VOC may bind to one or more sensors, and in the absence of a control cohort suffering from infection caused by viruses other than SARS‐CoV‐2 or by bacteria, it may be argued that the results are limited in their significance, and that biomarker identification would be the preferred option. However, considering the vast differences in the interindividual metabolism which is dependent on many factors such as body weight, nutrition, and other lifestyle factors such as stress, physical activity, or smoking habits, the quest to identify one specific molecule to reliably distinguish between infected and healthy subjects may be achievable only with extremely high subject numbers, if at all.

Instead, the breath gas analysis conducted here successfully and very rapidly provides information on the infectious state of the individual and could be comparable in its diagnostic relevance to other parameters obtained from blood analyses, for example, C‐reactive protein elevation, or clinical parameters such as elevated body temperature. Thus, the non‐invasive, inexpensive, and rapid result of E‐Nose technology‐based breath gas analysis may be used to warrant further, more invasive diagnostic measures to determine the exact underlying pathology.

ML technologies have been critically discussed as being instable, highly dependent of the training data and chosen analysis techniques, and potentially subject to cherry‐picking and a resulting over‐optimism of the reported results.^36^ In an effort to counteract these mechanisms and to assess the robustness of results, pseudonymized E‐Nose data of this study was made available to three independent teams of data analysts as part of a so‐called multi‐analyst experiment.

Experiments performed by team A indicated that breath analysis by E‐Nose technology as demonstrated here, allows for reliable discrimination between patients with SARS‐CoV‐2 infection and uninfected subjects. MEs were comparable to those of commercially available rapid antigen tests using oral or nasal swabs.^32^, ^37^ Analyzing feature importance revealed sensor 9 to be the most important for classification. Applying nested cross‐validation techniques ensured an unbiased estimation of the classification performance that would be obtained with independent data, while yielding results that are less impressive at first view.

Experiments of team B yielded an overview of the importance of features and time samples based on decision tree models. Again, sensor 9 had the highest impact on classification, and measurements from the first 10 s were the most informative. Experiments performed by team C again showed that models trained on data from sensor 9 were most effective at discriminating SARS‐CoV‐2 patients from uninfected subjects. Furthermore, exhaled air from the lower respiratory tract yielded better results than air obtained from the upper respiratory tract. A deep learning model could not outperform the more classical ML algorithms.

Adopting this multi‐analyst approach offers the advantage of combining a diverse range of skills, perspectives, and data analysis techniques that surpass what any individual analyst or research team could achieve. Moreover, the setting with several independent teams reduces the pressure on each team and thus also reduces the incentives to generate overly optimistic results through so‐called “fishing expeditions”—a term that commonly denotes the approach consisting of performing a large number of analyses until one of them yields a nice result, which is then often the result of advantageous random variations. Finally, a multi‐analyst experiment allows both to assess the analytical uncertainty associated with data analyses and, in many cases, including the study presented here, to achieve a consensus regarding the main results, which then can be considered robust.

In summary, the three teams used a variety of learners (decision tree, RF, gradient boosting, deep learning, elastic), data preprocessing, and evaluation procedures, illustrating the flexibility of analytical procedures in ML. They obtained different estimates of performance, which however were all in the range [0.06; 0.19]. Additionally, they all identified sensor 9 as the most informative sensor. This could provide a target for developing commercially attractive diagnostic tools. While previous studies show the potential feasibility to apply E‐Nose technology as a diagnostic tool,^17^ the present study generated an E‐Nose protocol for a quick, inexpensive, and statistically reliable detection of SARS‐CoV‐2 infection, with results similar to PCR tests and superior to POC antigen testing.^33^, ^34^, ^37^, ^38^

### 3.1 Strengths and limitations

This study was realized under challenging clinical conditions during the SARS‐CoV‐2 pandemic. The patients’ individual lifestyles, eating habits or potential undiagnosed diseases could not be entirely assessed and may be confounding factors. The limited sample size of *n* = 126 observations is another obvious limitation of this pilot study. Moreover, analytical choices such as the choice of the learner, parameter settings, preprocessing steps or the evaluation procedure can considerably influence the final results, as illustrated by the differences observed between the three teams. Other teams may have obtained different results with yet other approaches. However, despite of their differences, the results of the three teams also shared common components, such as the performance range or the importance of sensor 9. These findings can be considered robust and reliable. Finally, since every E‐Nose contains a differing, specific sensor set, different devices may lead to variations in readings for the same breath sample. This might potentially lead to the evaluation of a necessity of training the machine‐learning model for each specific E‐Nose setup.

## 4 METHODS

### 4.1 Study design and study subjects

In order to assess the diagnostic potential of the E‐Nose technology, we conducted a non‐interventional, non‐randomized, open prospective observational study. The aim was to discriminate between patients with viral SARS‐CoV‐2 infection upon admittance to the hospital and non‐infected subjects based on analysis of exhaled breath with an adapted E‐Nose technology approach. Patients who presented to the Emergency Department of the LMU Hospital, Munich, Germany, with signs of respiratory infection and who tested positive for SARS‐CoV‐2 by PCR were asked to participate in the study. Exclusion criteria were malignant diseases, treatment with immunosuppressants, pregnancy, age below 18 years, or inability to consent. As controls, non‐infected subjects were recruited. Non‐infected subjects were selected to match gender, age, height, weight, and smoking habits of the patient cohort (Table 1), and cohorts did not differ regarding lung disease or other metabolic comorbidities (Table S1).

Ethical approval was obtained from the LMU ethics committee (project no. 19−778), written informed consent was obtained from each subject, and the study was carried out in accordance with the Declaration of Helsinki.^39^

### 4.2 Breath and blood sampling

Breath samples were obtained from all patients on day one of their hospital stay and from all non‐infected subjects using the non‐invasive ReCIVA Breath Biopsy mask (Owlstone Medical) according to the manufacturer's instructions at least 1 h after the last meal or beverage. For sample collection, stainless steel Tenax TA sorption tubes (Markes International) were inserted into each pump's socket. A coated single‐use sterile silicon mask (Owlstone Medical) was used for each subject. Medical grade oxygen with a flow rate of 35−40 L/min was administered via the ReCIVA device to reduce resistance and to enable normal breathing. Breath cycle patterns were detected by built‐in CO_2_ and pressure sensors and analysed in real time by the Breath Sampler Controller software v3.4 (Owlstone Medical) run on a tablet computer (Microsoft Surface Pro 7, Microsoft Corporation). Different pumps with separate Tenax TA tubes for breath collection were activated accordingly during the initial or later phase of expiration to obtain samples from the upper or lower respiratory tract, respectively. For each sample, 500 mL of exhaled breath was collected at a flow rate of 250 mL/min. Tubes were then capped and stored for a maximum of 2 h until analysis.^40^, ^41^ The equipment was disinfected with ethanol 70% to prevent microbial contamination or contamination with VOC emission from commercially available disinfectants.

### 4.3 VOC analysis by metal oxide E‐Nose technology

Tubes were decapped, inserted into the Enrichment and Desorption Unit (EDU, AIRSENSE Analytics GmbH) and the measurement cycle was started using the WinMuster software v1.6.2.25 (AIRSENSE Analytics GmbH). This pre‐analytic step was chosen, as concentrations of some VOC in human breath may be too low to be detected by a regular E‐Nose setup. Application of an EDU can achieve enrichment factors of up to 1000, and additionally depletes the sample of water vapors, allowing for VOC measurement at a lower concentration range. Tubes were heated to 220°C to dissolve VOC from the adsorption resin. After 160 s, the air sample was flushed through the transfer line into the Portable Electronic Nose (PEN3.5, AIRSENSE Analytics GmbH), allowing for binding of VOC to an array of 10 metal oxide semiconductors, temporarily altering sensor conductivity. Specifics of these sensors are provided in Table S3. Changes in conductivity were measured for 60 s and depicted color‐coded for each sensor. After analysis, sorption tubes were removed from the EDU, conditioned with a thermal desorber (TC‐20, Markes International) at 280°C for 20 min according to the manufacturer's instructions, capped, and stored for further use.

Routine blood analyses upon admission were performed for blood cell count and inflammation markers. EDTA‐anticoagulated samples were drawn by peripheral venous puncture and analyses were carried out by the Institute of Laboratory Medicine, LMU University Hospital, Munich, Germany.

### 4.4 Data collection and format

**FIGURE 6:**
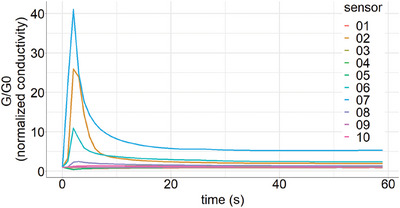
Exemplary signal pattern generated by E‐Nose analysis. Curves depict changes in conductivity of 10 different metal oxide semiconductor sensors over time. Each line indicates one sensor. Conductivity (*G*) is normalized for each sensor's zero value (*G*0) measured before injection.

Raw data consisted of one time series for each metal oxide sensor of the E‐Nose, that is, 10 sensors resulting in 10 time series for 60 s for each individual. Measurements were available for both upper and lower respiratory tract samples, resulting in a total of 20 time series per individual over 60 s. A binary label indicated if the individual was SARS‐CoV‐2 negative or positive. Figure 6 shows a graphical depiction of raw E‐Nose data obtained from one representative measurement.

### 4.5 Data analyses

**FIGURE 7:**
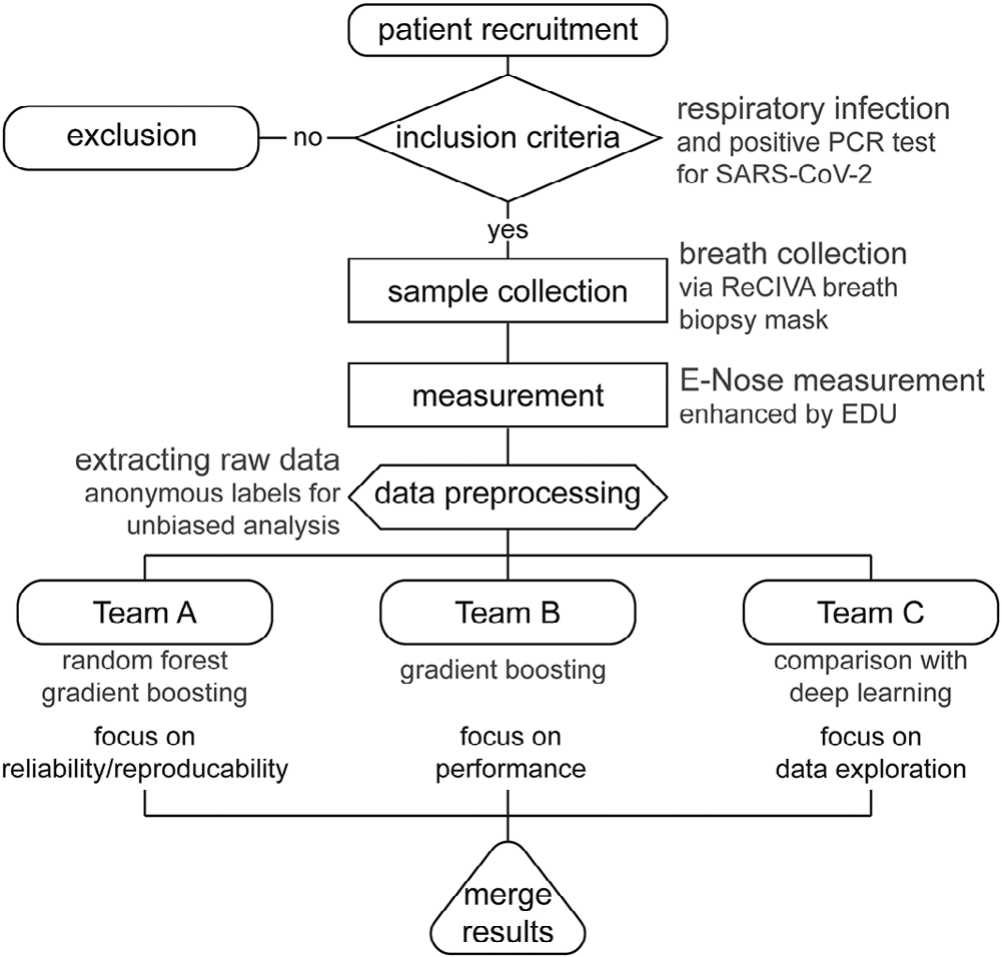
Study design and multi‐analyst approach. Schematic depiction of the study workflow and comparison of different analytical approaches and foci of data processing. PCR, polymerase chain reaction; EDU, Enrichment and Desorption Unit; SARS‐CoV‐2, severe acute respiratory syndrome coronavirus type 2.

Raw conductivity data were extracted into transfer files, which were then subjected to analysis by three independent parties. In an effort to provide unbiased results, these three teams did not consult each other, thus allowing different impartial choices of analytic approaches. Team A was from the LMU Institute for Medical Information Processing, Biometry and Epidemiology (IBE) with vast experience in medical data processing. Team B was the Artificial Intelligence Company LANZ GmbH, and team C consisted of space physics experts at Airbus Defence and Space GmbH familiar with E‐Nose raw data processing (Figure 7). Experiments were numbered consecutively from I to VII. Details for the analytical approaches of the three teams are provided as Supporting Information. Results were reported with varying figures of merit, which were transformed to MEs for better comparability. The lead team refrained from conducting an analysis to ensure non‐biased reporting of independent results. All of the results are reported and none were excluded.

## AUTHOR CONTRIBUTIONS

*Conceptualisation, data curation, investigation, methodology, supervision, validation, visualisation, writing—original draft, and writing—review and editing*: T.W. and A.C. *Data curation, formal analysis, investigation, methodology, visualisation, writing—original draft, and writing—review and editing*: F.P. *Formal analysis, investigation, methodology, supervision, validation, visualisation, writing—original draft, and writing—review and editing*: M.M.M. *Investigation, methodology, supervision, writing—original draft, and writing—review and editing*: A.L.B. *Formal analysis, investigation, methodology, validation, visualisation, writing—original draft, and writing—review and editing*: W.S., J.H., A.K., and D.L. *Conceptualisation, methodology, writing—review and editing*: M.F., M.D., M.K., and B.A. *Investigation, methodology, and writing—review and editing*: L.K. and D.M. All authors read and approved the final manuscript.

## CONFLICT OF INTEREST STATEMENT

A.K. and J.H. are employees of Airbus Defense & Space, and authors W.S. and D.L. are part of Lanz GmbH, and have no potential relevant financial or non‐financial interests to disclose. The other authors have no conflicts of interest to declare.

## ETHICS STATEMENT

Ethical approval was obtained from the LMU ethics committee (project no. 19‐778), written informed consent was obtained from each subject, and the study was carried out in accordance with the Declaration of Helsinki.

## Supporting information



Supporting Information

## Data Availability

The datasets used and analyzed during the current study are available from the corresponding author on reasonable request.
