# Supplementary material for: Point‐of‐care breath sample analysis by semiconductor‐based E‐Nose technology discriminates non‐infected subjects from SARS‐CoV‐2 pneumonia patients: a multi‐analyst experiment
Source: MedComm (2020). 2024 Oct 24;5(11):e726. doi: 10.1002/mco2.726 (PMC11502717; doi:10.1002/mco2.726)
Supplement: Supplementary file 1 — Supporting Information [file MCO2-5-e726-s001.pdf]

Supplementary Tables and Figures

Table S1

	**COV** (n=63)	**CTRL** (n=63)	***p* value**
Obesity	24	15	.0841[a]
Hypothyreoidism	8	9	.7963[a]
Arterial hypertension	22	14	.1165[a]
Diabetes mellitus type I	2	0	.1590[a]
Diabetes mellitus type II	7	2	.0856[a]
Bronchial asthma	7	2	.0856[a]
COPD	1	0	.3212[a]

Table S1. Comorbidities in SARS-CoV-2 positive patients and controls. The cohorts showed no significant difference with regard to lung disease or other metabolic diseases. [a]Student's *t*-test. COPD, chronic obstructive pulmonary disease. COV, SARS-CoV-2 positive cohort; CTRL, controls.

Table S2

	**respiratory tract**	**median**	**IQR**
**ground truth**<br>(sum of FP of both cohorts)	lower<br>upper	0<br>1	0-2<br>0-3
**'real' leave-one-out**<br>(sum of FP of both cohorts)	lower<br>upper	11<br>13	10-11<br>12-13

Table S2. Ground truth vs 'real' leave-one-out test. 26 classifiers (52%) were able to classify all subjects correctly during the ground truth experiment. The remaining 24 classifier variations were not able to identify all subjects correctly even when the subject was included in the training data. FP; false positives, IQR; interquartile range.

Table S3

sensor number	sensor name	general description	calibration gas	temperature (°C)
1	W1W<br>aromatic	aromatic compounds	benzene 10 ppm<br>ethanol 20 ppm	400
2	W5S<br>broadrange	broad range sensitivity<br>very sensitive, with negative signal	benzene 10 ppm<br>H_2S 1 ppm	350
3	W3C<br>aromatic	ammonia, aromatic compounds	benzene 10 ppm<br>ethanol 20 ppm	450
4	W6S<br>hydrogen	mainly hydrogen	-	450
5	W5C<br>arom-aliph	alkanes, aromatic compounds,<br>less polar compounds	benzene 10 ppm<br>ethanol 20 ppm	500
6	W1S<br>broad-methane	broad range, sensitive to methane,<br>similar to sensor 8	CH_4 10 ppm	400
7	W1W<br>sulphur-organic	sulphur compounds (H_2S 0.1 ppm)<br>sensitive to terpenes, sulphur organic compounds	H_2S 1 ppm	450
8	W2S<br>broad-alcohol	broad range, detects alcohols,<br>partially aromatic compounds	benzene 10 ppm<br>ethanol 20 ppm	350
9	W2W<br>sulphur-chlor	aromatic compounds, sulphur organic compounds	H_2S 1 ppm	500
10	W3S<br>methane-aliph	concentrations > 100 ppm, selective for methane	CH_4 10 ppm	400

Table S3. **Characterization of the E-Nose sensor array.** Sensitivities and operation parameters of the 10 metal oxide semiconductor sensors installed in the Portable Electronic Nose PEN3.5. Sensors were selected to detect a wide range of VOC exhaled in human breath. Sensor specifics were provided by the manufacturer. ppm; parts per million.

Table S4

- mlr3verse (0.2.2)
- mlr3 (0.13.0)
- mlr3learners (0.5.0)
- mlr3tuningspaces (0.3.0)
- mlr3tuning (0.13.1)
- mlr3filters (0.5.0)
- iml (0.10.1)
- tsfeatures (1.0.2)
- stringr (1.4.0)
- dplyr (1.0.8)
- rlist (0.4.6.1)
- ranger (0.12.1)

Table S4. Libraries used by Team A. For more detailed instructions please refer to the readme-file in the online appendix.[47]

Figure S1

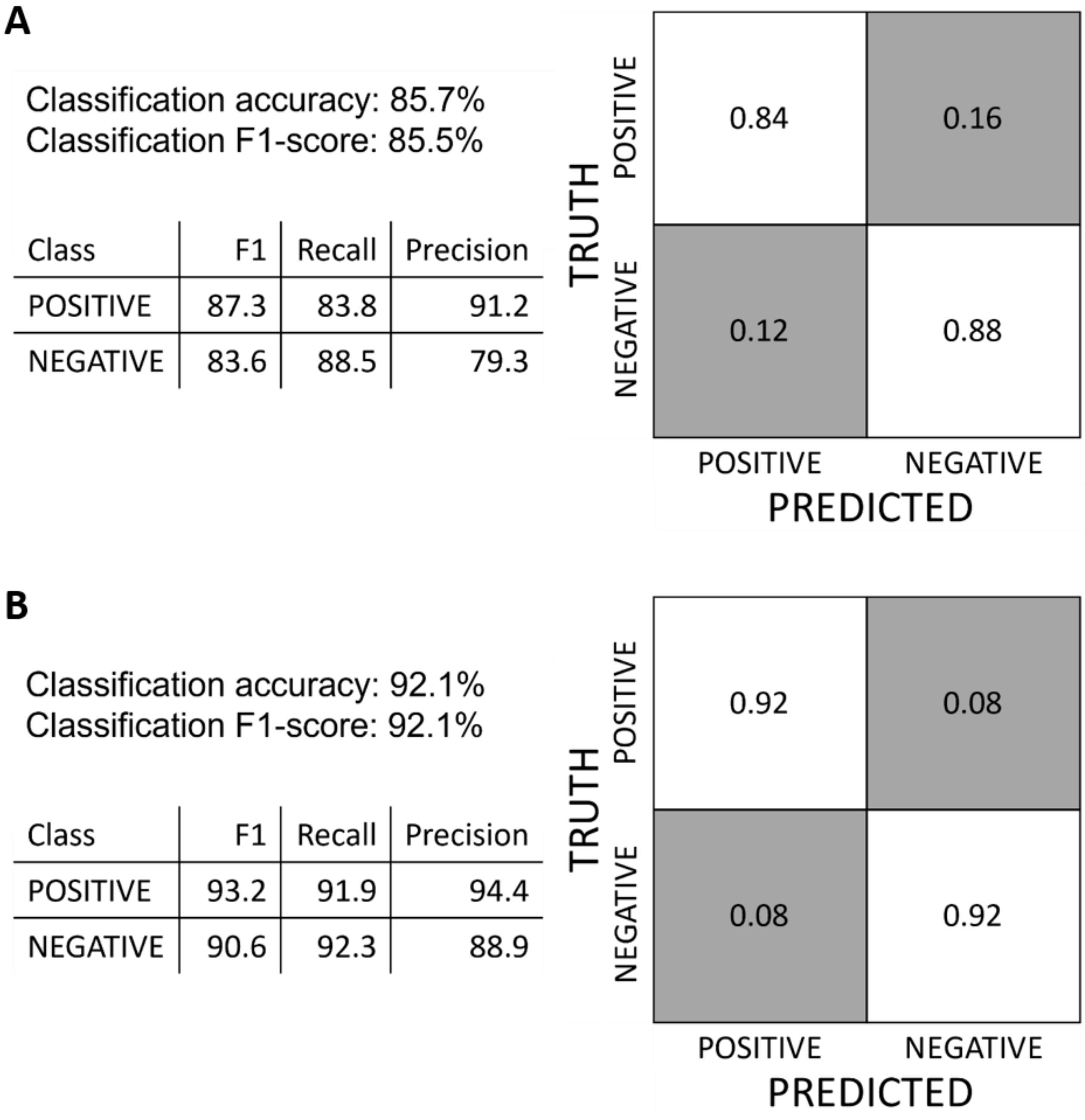

Figure S1. Classification accuracy, F1-score results, and confusion matrix for experiments III and IV. LightGBM‘s gradient boosting decision tree learner trained on data from all sensors and both upper and lower respiratory tract samples without and with hyperparameter optimization, achieved an overall classification accuracy of 85.7% (**a**) and 92.1% (**b**), respectively.

Figure S2

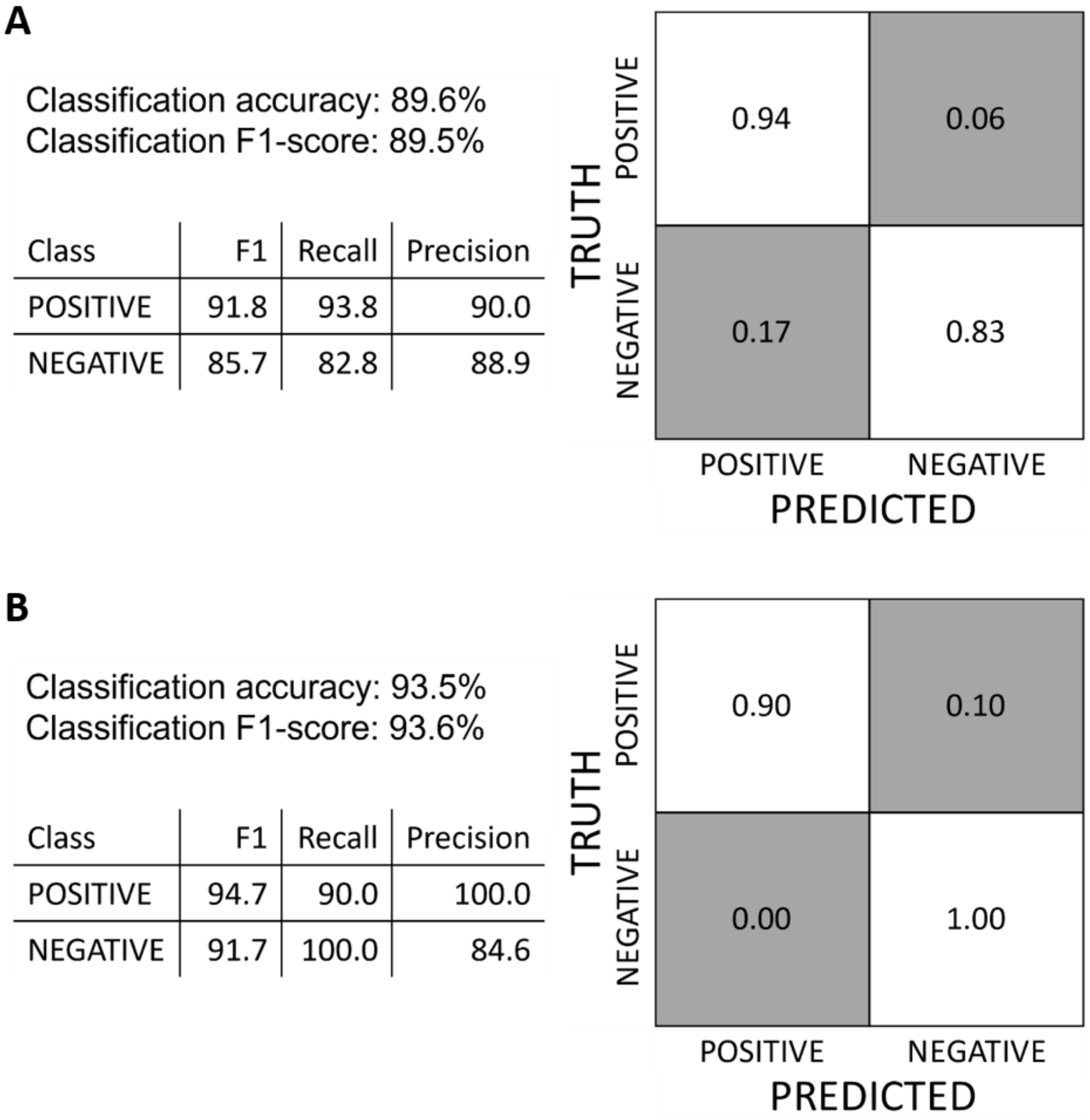

Figure S2. Classification accuracy, F1-score results, and confusion matrix for experiments V and VI. Compared to the previous experiments, LightGBM‘s gradient boosting decision tree learner trained on data from only sensor 9 outperformed learners trained on data from all sensors with a classification accuracy of 89.6% without (**a**) and 93.5% with hyperparameter optimization (**b**).

Figure S3

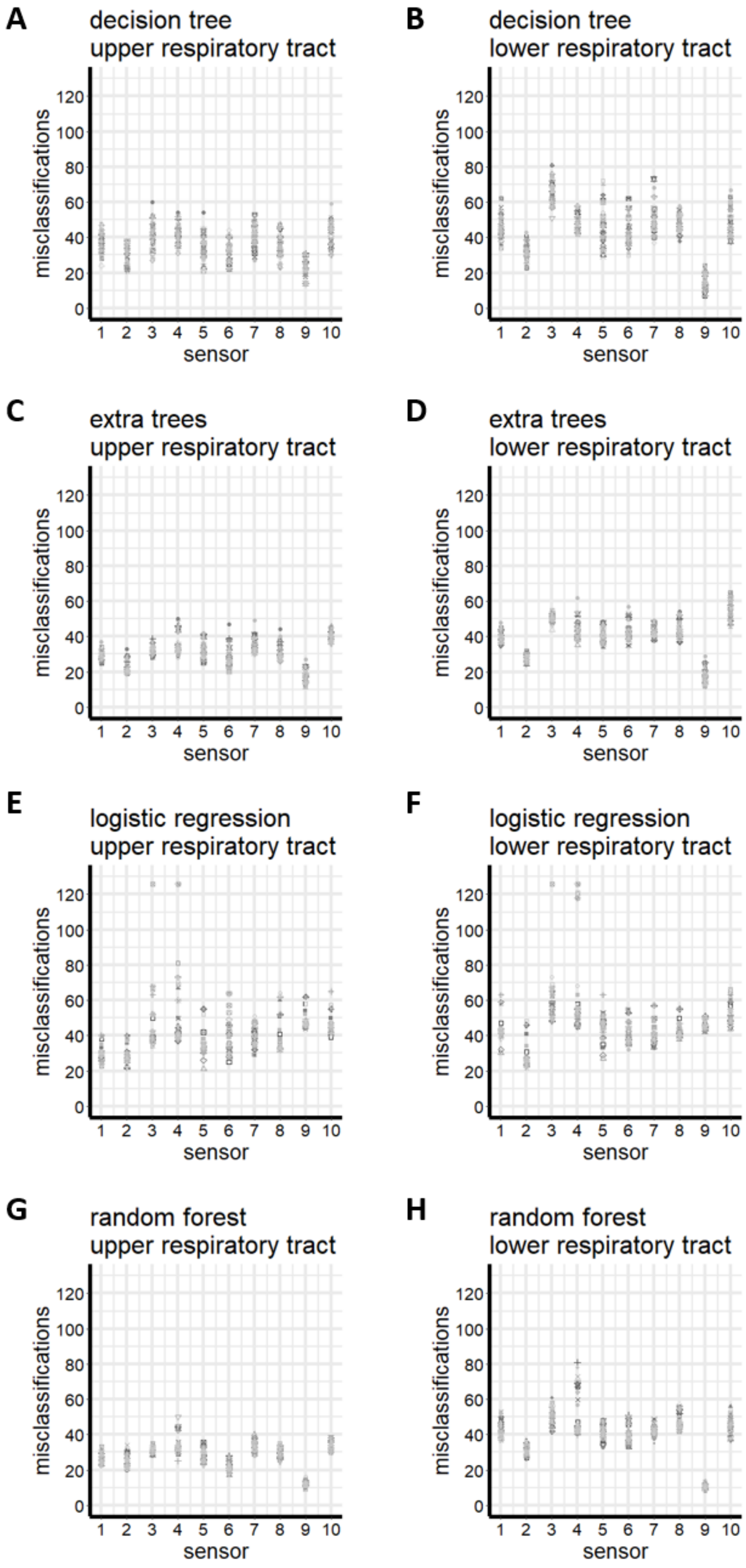

Figure S3. Sum of false positives of both infected and uninfected cohorts for 50 hyperparameter sets over all sensors and classifiers. Classifier random forest (RF) showed the best results of all classifiers, and data from sensor 9 from lower respiratory tract samples for all sensors. Thus, all further analyses were performed with classifier RF and sensor 9 only.

Figure S4

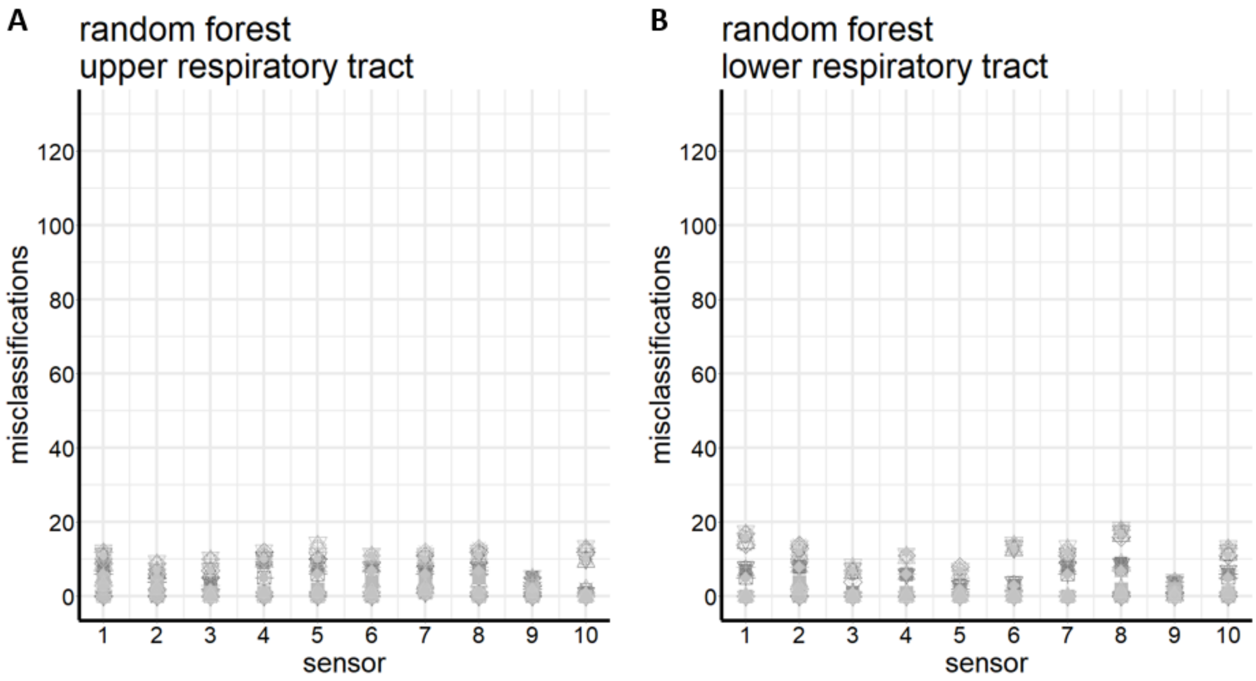

Figure S4. Sum of false positives of both infected and uninfected cohorts for 50 hyperparameter sets over all sensors for random forest classifier ground truth experiment. Not all subjects were identified correctly, although the score was much lower than for the real leave-one-out test. Again, sensor 9 showed superior performance regarding hyperparameter sensitivity for both upper (**a**) and lower (**b**) respiratory tract samples.

Figure S5

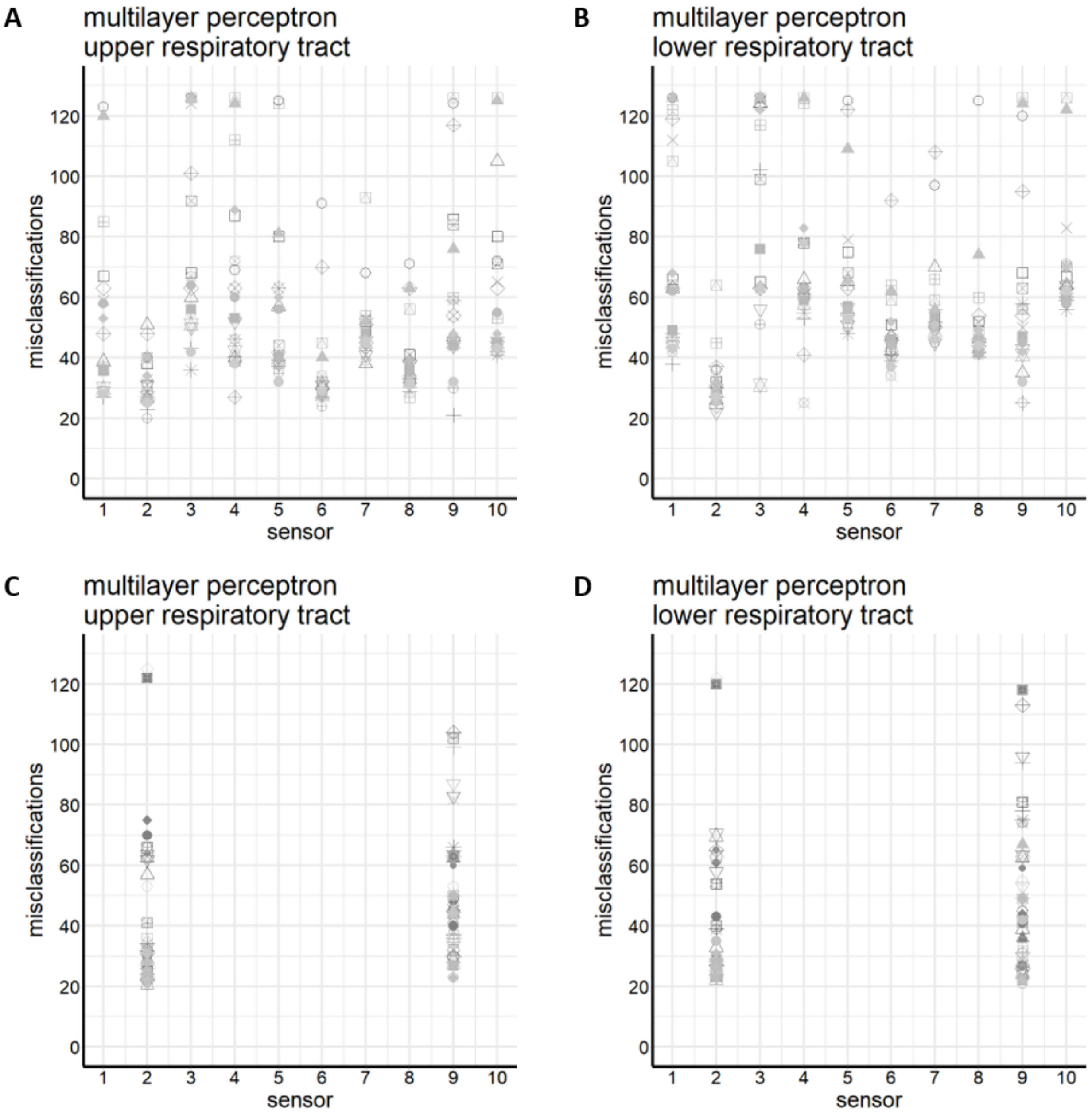

Figure S5. Sum of false positives of both infected and uninfected cohorts for multilayer perceptron classifier. For MLP, sensors 2 and 9 also perform best for both upper and lower respiratory tract samples, but variation regarding hyperparameters is higher than with previous classifiers (**a, b**). Still, MLP could not outperform classifier RF, even with adapted hyperparameters (**c, d**).

Supplementary Information

Methods

Team A

Data pre-processing

Two different analyses were conducted by Team A. Experiment I used raw data and did not consider time dependencies of time-structured data. Accordingly, this resulted in 1200 features consisting of features for every second for each sensor (lower respiratory tract: 60 seconds x 10 sensors; upper respiratory tract: 60 seconds x 10 sensors).

Experiment II used feature extraction to create a number of features describing each time series, namely stability, lumpiness, standard deviation, fluctanal, minimum, and maximum of each time series. Stability is defined as the variance of means of the time series based on non-overlapping windows with predefined length, while lumpiness is the variance of variances of these non-overlapping windows. Fluctanal fits a polynomial of order 1 to the time series and returns its range.[42,43] Minimum, maximum, and standard deviation return the minimal value, maximal value, and standard deviation, respectively, of the entire time series. Compared to experiment I, methods applied in experiment II aimed at giving more consideration to the time structure of the raw data. This resulted in a total of 120 features: 6 measures for 10 sensors for both, the lower and the upper respiratory tract.

Performance Evaluation

For the final performance evaluation, a nested resampling procedure[44] was implemented on both a random forest (RF, a data-driven, complex machine learning algorithm) and a generalized linear model with elastic net regularization (Glmnet, representing an easier, interpretable model class). Nested resampling was performed with a 7-fold cross-validation (CV) in the inner loop and 5-fold CV in the outer loop for performance evaluation. More precisely, data was split into five ‘outer’ folds, resulting in five pairs of training/test sets. For both considered learners the following procedure was applied: on each of the 5 outer training sets hyperparameter tuning was performed through an ‘inner’ 7-fold CV. For each outer CV iteration, this returned a set of selected hyperparameter values, which was then used to fit the considered learner to the considered outer training set. The performance of the resulting model was then evaluated on the corresponding outer test set based on the AUC and the classification error. Performance was finally averaged over the five outer CV iterations. For tuning the respective hyperparameters (RF: mtry.ratio, sample.fraction, num.trees/Glmnet : alpha, lambda), a random search with a maximum of 1,000 evaluations each was implemented.[45]

For the assessment of feature importance two approaches were implemented: (1) an approach based on the mean decrease in impurity[46] and (2) the ad-hoc interpretable coefficients of the generalized linear model. These measures are based on the tuned final model fitted on the whole dataset.

Implementation and Reporting

The models were implemented using R version 4.0.4 (2021-02-15) on a x86_64-pc-linux-gnu (64-bit) platform running under Debian GNU/Linux 11 (bullseye). For reproducibility, libraries used by Team A are listed in **Table S4.** For more detailed instructions including the analysis code, please refer to our GitHub repository (https://github.com/mmax-code).[47]

Team B

Data pre-processing

Data was read, and a flat mapping was created where each array entry represents a single file with all additional meta information (folder name, file name, date, domain knowledge about the sample) and raw sensor data. Results were saved in JSON format for fast and native handling in either Python or JavaScript environments. Each segment was normalized in [0,1] range using the absolute maximum of contained samples.

Performance Evaluation

An iterative configure/train/validation/test loop was used to explore model behavior with respect to different dataset sizes, filtering techniques and general hyperparameter settings. After obtaining baseline results, the stepwise hyperparameter tuning approach from Optuna (https://github.com/optuna/optuna, version 2.10.0) was used. Unless stated otherwise, a default train/test split of 2:1 was chosen. Splitting was carried out randomly, and samples were shuffled. Random splitting seeds were stored for future reproducibility. Average accuracy, average F1 score, class F1 score, class recall, and class precision were selected as output metrics. A confusion matrix was generated for each model. Experiments III and IV included data from all sensors, experiments V and VI used data obtained from sensor 9 only.

Implementation and Reporting

A Jupyter notebook environment in python was used for all steps involved, from data processing to training, evaluation, and visual representation. For machine learning, LightGBM's (https://github.com/Microsoft/LightGBM, version 3.3.1) gradient boosting model was used.

Team C

Data pre-processing

It was assumed that the information from which cohort a sample was taken is encoded in the shape of the measurement's time series and that each point of the timeline could contribute equally to the classification success. It could not be excluded that the available samples were unrepresentative, or that an attempt to identify the most significant features of the time series could be prone to systematical errors caused by statistical fluctuation. Thus, it was decided to use the unprocessed time series and to treat every measured value as a feature, irrespective of its correlation with neighboring values.

Performance evaluation

First, sensors and classifiers with the best classification performance were identified. As a figure of merit, the sum of false classifications of both cohorts was chosen. If this figure is small, sensitivity and specificity are high, which leads to high accuracy. Sensitivity and specificity were calculated using the leave-one-out method. Every subject was classified with a classifier that was trained on the entire dataset, with the subject to be tested excluded from this dataset. Hyperparameters were varied 50 times for the classifiers extra tree (ET), decision tree (DT), random forest (RF), and logistic regression (LR) (**Figure S3**).

Classifier evaluation was complemented by testing if the learner was able to correctly classify all subjects if the subject to be tested is contained in the training data (ground truth). Again, hyperparameters were varied 50 times to capture the effect of classifier configuration.

For hyperparameter optimization, sensor 9 and classifier RF were chosen, hyperparameters were randomly varied 100 times to improve results. As figures of merit, sensitivity and specificity were chosen, derived from the true positive and true negative counts (experiment VII).

It was furthermore examined how the amount of training data influenced precision and negative predictive value. Classifier RF was trained with the best set of hyperparameters using 25%, 50%, 75% and 100% of training data.

Experimentation with deep learning models

In addition to the more classical machine learning approaches, a deep learning model was also tested. For this purpose, a multilayer perceptron (MLP) was used for classification. Hyperparameters were varied randomly 20 times and all sensors were classified using leave-one-out.

Graphical Abstract and graph design

BioRender software was used to create the Graphical Abstract (www.BioRender.com, licence to A.C.), and graph design was performed with RStudio (Boston, MA, USA. URL http://www.rstudio.com) and Adobe Illustrator (Adobe Inc., DE, USA. URL https://adobe.com/products/illustrator).

REFERENCES:

42. Fulcher BD, Jones NS. hctsa: A Computational Framework for Automated Time-Series Phenotyping Using Massive Feature Extraction. *Cell Syst*. 2017; **5**:527-31 e3.
43. Fulcher BD, Little MA, Jones NS. Highly comparative time-series analysis: the empirical structure of time series and their methods. *J R Soc Interface*. 2013; **10**:20130048.
44. Varma S, Simon R. Bias in error estimation when using cross-validation for model selection. *BMC Bioinformatics*. 2006; **7**:91.
45. Bischl B, Binder M, Lang M, Pielok T, Richter J, Coors S, et al. Hyperparameter optimization: Foundations, algorithms, best practices, and open challenges. *WIREs Data Mining and Knowledge Discovery*. 2023; **13**:e1484.
46. Breiman L, Friedman J, Stone CJ, Olshen RA. Classification and Regression Trees: Taylor & Francis; 1984.
